# The W101C *KCNJ5* Mutation Induces Slower Pacing by Constitutively Active GIRK Channels in hiPSC-Derived Cardiomyocytes

**DOI:** 10.3390/ijms242015290

**Published:** 2023-10-18

**Authors:** Anne Kayser, Sven Dittmann, Tomo Šarić, Giulia Mearini, Arie O. Verkerk, Eric Schulze-Bahr

**Affiliations:** 1 Institute for Genetics of Heart Diseases (IfGH), University Hospital Münster, 48149 Münster, Germanysven.dittmann@ukmuenster.de (S.D.); eric.schulze-bahr@ukmuenster.de (E.S.-B.); 2 Center for Physiology and Pathophysiology, Institute for Neurophysiology, Faculty of Medicine and University Hospital Cologne, University of Cologne, 50931 Cologne, Germany; tomo.saric@uni-koeln.de; 3 Institute of Experimental Pharmacology and Toxicology, University Medical Center Hamburg-Eppendorf, 20246 Hamburg, Germany; 4 Department of Medical Biology, Amsterdam University Medical Centers, University of Amsterdam, 1105 AZ Amsterdam, The Netherlands; 5 Department of Experimental Cardiology, Amsterdam University Medical Centers, University of Amsterdam, 1105 AZ Amsterdam, The Netherlands

**Keywords:** *KCNJ5*, sinus node dysfunction, hiPSC, cardiomyocytes, disease model, I_K,ACh_ blocker XAF-1407

## Abstract

Mutations in the *KCNJ5* gene, encoding one of the major subunits of cardiac G-protein-gated inwardly rectifying K^+^ (GIRK) channels, have been recently linked to inherited forms of sinus node dysfunction. Here, the pathogenic mechanism of the W101C *KCNJ5* mutation underlying sinus bradycardia in a patient-derived cellular disease model of sinus node dysfunction (SND) was investigated. A human-induced pluripotent stem cell (hiPSCs) line of a mutation carrier was generated, and CRISPR/Cas9-based gene targeting was used to correct the familial mutation as a control line. Both cell lines were further differentiated into cardiomyocytes (hiPSC-CMs) that robustly expressed GIRK channels which underly the acetylcholine-regulated K^+^ current (I_K,ACh_). hiPSC-CMs with the W101C *KCNJ5* mutation (hiPSC^W101C^-CM) had a constitutively active I_K,ACh_ under baseline conditions; the application of carbachol was able to increase I_K,ACh_, further indicating that not all available cardiac GIRK channels were open at baseline. Additionally, hiPSC^W101C^-CM had a more negative maximal diastolic potential (MDP) and a slower pacing frequency confirming the bradycardic phenotype. Of note, the blockade of the constitutively active GIRK channel with XAF-1407 rescued the phenotype. These results provide further mechanistic insights and may pave the way for the treatment of SND patients with GIRK channel dysfunction.

## 1. Introduction

Sinus node dysfunction (SND) is clinically manifested in sinus bradycardia, chronotropic incompetence or sinus arrest as a cause of disturbed cardiac impulse generation, delayed or absent cardiac conduction between sinoatrial node (SAN) and atria [1]. Depending on the pathogenesis, SND can classified into primary or secondary SND [2]. Patients with SND show a broad range of clinical symptoms, e.g., dizziness, syncope, fall [3], and it may increase the risk of cardiac arrhythmias [4]. SND may be due to extrinsic or intrinsic factors and both exhibit variable manifestations, as recently reviewed by Sathnur and colleagues [5]. It was increasingly recognized that ion channel dysfunction and inherited forms of so-called channelopathies are an important cause for intrinsic and familial SND resulting in an upcoming list of ion-channel genes being involved in SND (for reviews, see [4,6,7,8]). Among them, the p.Trp101Cys (short: W101C) mutation in the *KCNJ5* gene has recently been identified in a family with SND [9]. *KCNJ5* encodes the G-protein-gated inwardly rectifying K^+^ channel Kir3.4 (GIRK4), which is mainly expressed in the heart, pancreas, and in parts of the adrenal gland [10,11,12]. Kir3.4 forms heterotetramers with Kir3.1 (GIRK1, encoded by *KCNJ3*), which results in the acetylcholine-activated G-protein-gated inwardly rectifying K^+^ (GIRK) channels which underly the K^+^ current, I_K,ACh_ [4,13], that is present in the human SAN and atria. The activation of GIRK channels, resulting in an increased I_K,ACh_, elongates the intrinsic cycle length of SAN cells and hyperpolarizes the maximal diastolic membrane potential (MDP) in SAN and atrial cardiomyocytes [14,15,16,17,18]. In addition to the high-level protein expression of GIRK4 subunits in atrium, a higher expression was also detected in the hypothalamus [19]. Here, it was especially found in the paraventricular nucleus of the cardiac vagal neurons, which may play an additional role in heart-rate control and cardiac function [20].

Currently, 31 disease-causing variants in the *KCNJ5* gene are known to date. Potential heart disease variants in *KCNJ5* have been described and may be causative for long QT syndrome, Andersen-Tawil syndrome, atrial fibrillation, short QT syndrome, or unspecified events like sudden cardiac arrest or sudden unexpected death [21,22,23,24,25,26,27,28,29]. However, due to the small number of cases and mostly missing functional data, *KCNJ5* is not a proven gene for the respective diseases. In addition, mainly somatic variants have been associated with primary aldosteronism and hypertension. The *KCNJ5* variant W101C has been reported as a gain-of-function variant in familial SND [9]; functional data in heterologous systems, e.g., CHO cells and *Xenopus* oocytes, demonstrated a constitutively active I_K,ACh_ [9]; thus far, data in human-derived cardiomyocytes disease models are lacking. Here, we generated patient-specific (from a *KCNJ5* W101C mutation carrier) induced pluripotent stem cells (hiPSC) and an isogenic control cell line via CRISPR/Cas9. Cardiomyocytes derived from these hiPSC lines (hiPSC-CMs) were electrophysiologically analyzed by patch-clamp techniques. We found that hiPSC-CMs with the mutant GIRK channel had a slowed pacemaking activity due to a constitutive channel activation and that I_K,ACh_ channel inhibition might pave a way for potential targets for SND treatment.

## 2. Results

### 2.1. Generation and Genetic Correction of hiPSCs from a Patient with the W101C KCNJ5 Variant

**Figure 1:**
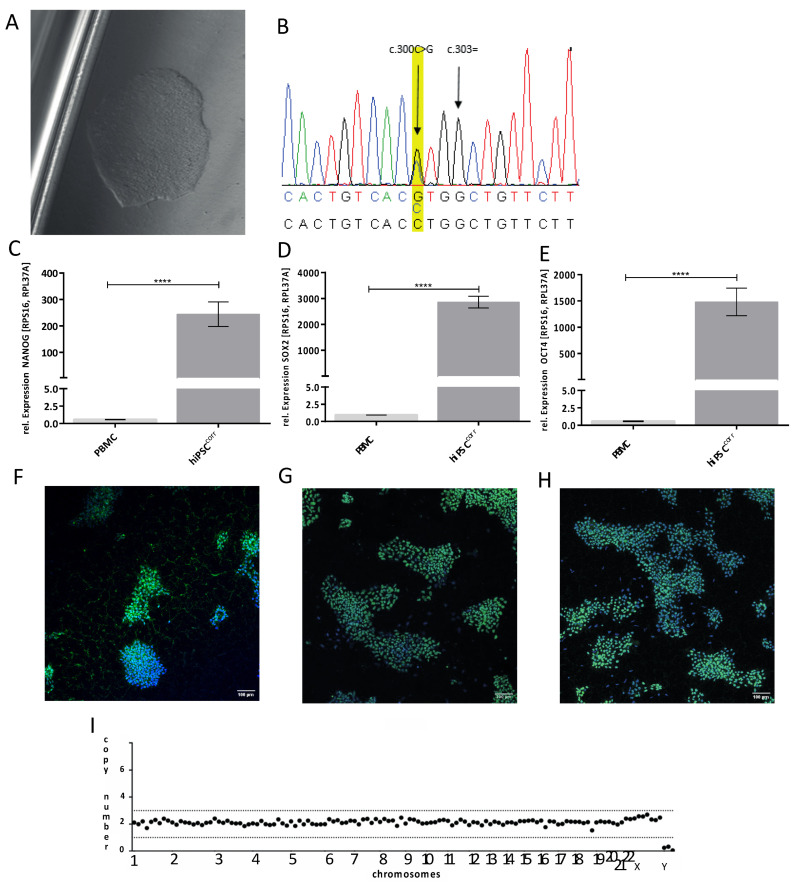
Characterization of the hiPSC^corr^ line. (**A**) Brightfield microscopy picture of a corrected *KCNJ5* W101C hiPSC clone (hiPSC^corr^). (**B**) Sanger sequencing results after correction of the pathogenic *KCNJ5* W101C variant. The nucleotide exchange at position c.303 was successfully corrected and a silent mutation for protection of the HDR-repair template (c.300C>G) was inserted. (**C**–**E**) NANOG, SOX2, and OCT4 as pluripotency markers on RNA level via RT-qPCR. **** = *p* < 0.0001. (**F**–**H**) Immunofluorescence staining of NANOG (green), SOX2 (green), and OCT4 (green). The nucleus is stained with DAPI (blue). (**I**), Molecular karyogram of corrected *KCNJ5* W101C hiPSC (hiPSC^corr^). No chromosomal abrasions were detected.

For characterization of mutant and isogenic corrected I_K,ACh_ channels, first patient-specific peripheral blood mononuclear cells (PBMCs) were isolated via density gradient centrifugation with Ficoll™ and frozen at −80 °C until further use. The reprogramming of these PBMCs into stem cells was carried out via Sendai-virus-based vectors, as set out in detail in Section 4.1. The cell clone KCNJ5 K8 was used for this study and registered in the Human Pluripotent Stem Cell Registry (hPSCreg^®^ number: UKMi005-A). In addition, an isogenic control hiPSC line (hiPSC^corr^) was generated with CRISPR/Cas9-mediated genome editing, as described in Section 4.3. Figure 1A shows a brightfield microscopy image of the control *KCNJ5* hiPSC line (hiPSC^corr^), and Figure 1B demonstrates the successful correction of the pathogenic *KCNJ5* W101C variant as addressed by Sanger sequencing. The hiPSC^corr^ line maintained expression of NANOG, SOX2, and OCT4 on the RNA level and protein level (Figure 1C–H), and CRISPR/Cas9-mediated genome editing had no effect on the karyotype (Figure 1I). This cell line is also registered at hPSCreg^®^ (number: UKMi006-A).

### 2.2. Variant W101C KCNJ5 Results in a Constitutively Active I_K,ACh_ Current after Retinoic Acid (RA)-Based Differentiation into Atrial-like hiPSC^W101C^-CMs

To study the effect of the W101C variant on I_K,ACh_, isogenic hiPSC^corr^ and hiPSC^W101C^ lines were next differentiated into cardiomyocyte-like cells (hiPSC-CMs). Hence, a retinoic acid (RA)-based differentiation protocol was used, from which it is well-known that they promote the acquisition of atrial-like features and with robust expression of *KCNJ3* as an atrial marker (Supplement Figure S1C) [30,31,32,33,34,35,36]. Such hiPSC-CMs further enable electrophysiological studies of mutations involved in I_K,ACh_ channel activation [32].

**Figure 2:**
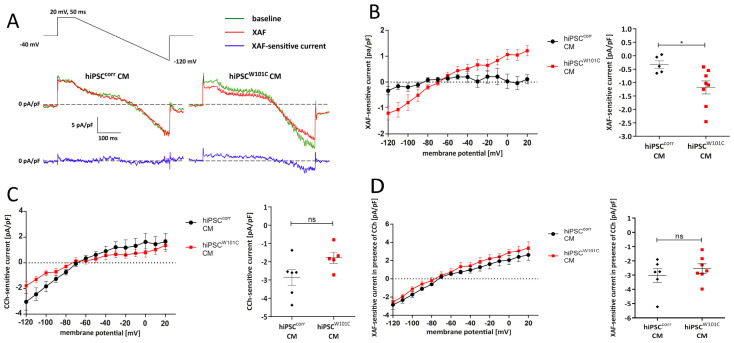
I_K,ACh_ densities in isogenic RA-treated hiPSC^corr^-CMs and hiPSC^W101C^-CMs. (**A**) Typical ramp traces in the absence and presence of XAF-1407 (XAF), a selective I_K,ACh_ channel locker, in hiPSC^corr^-CMs and hiPSC^W101C^-CMs. (**B**) Current–voltage (I–V) relationships of XAF-sensitive currents in hiPSC^corr^-CMs (black) and hiPSC^W101C^-CMs (red) under baseline conditions (**left panel**) and dot plots of the XAF-sensitive currents at −120 mV (**right panel**). * = *p* < 0.05. (**C**) I–V relationships of CCh-sensitive currents (**left panel**) and dot plots of CCh-sensitive currents at −120 mV (**right panel**). ns = not significant. (**D**) I–V relationships of XAF-sensitive currents in the presence of CCh (**left panel**) and dot plots of XAF-sensitive currents in the presence of CCh at −120 mV (**right panel**). ns = not significant.

First, it was tested whether the W101C *KCNJ5* variant results in constitutively activated I_K,ACh_ channels, similarly as previously shown in a heterologous cell expression system [9]. Therefore, we tested if the new developed highly-selective I_K,ACh_ current inhibitor XAF-1407 [37,38,39] reduces I_K,ACh_ currents under baseline conditions during a descending voltage-clamp ramp (see Section 4.9). Figure 2A,B shows typical recordings and average current–voltage (I–V) relationships, respectively, in hiPSC^corr^-CMs and hiPSC^W101C^-CMs. The results show that XAF-sensitive currents were virtually absent in hiPSC^corr^-CMs (Figure 2A,B). However, in hiPSC^W101C^-CMs, an XAF-sensitive current was clearly present at baseline conditions (Figure 2A,B). For example, the average current density of the XAF-sensitive current in hiPSC^W101C^-CMs at −120 mV was −1.2 ± 0.2 pA/pF (n = 8), which is significantly larger than in hiPSC^corr^-CMs (Figure 2B). Thus, the W101C *KCNJ5* mutation results in a constitutively active I_K,ACh_ current in hiPSC-CMs.

Secondly, it was analyzed whether all mutant GIRK channels were constitutively open due to the presence of the W101C mutation under baseline conditions and whether carbachol (CCh), a structure analog of acetylcholine and agonist of the muscarinic receptor, could open additional channels. By applying 10 µM CCh, a robust I_K,ACh_ current could be evoked in both hiPSC^corr^-CMs and hiPSC^W101C^-CMs (Figure 2C). There was a tendency of larger CCh-activated I_K,ACh_ in hiPSC^corr^-CMs (Figure 2C), but this was finally not significant (*p* = 0.08; unpaired *t*-test). In a third voltage clamp experiment, the total I_K,ACh_ density, i.e., constitutively active I_K,ACh_ plus CCh-activated I_K,ACh_, was tested between hiPSC^corr^-CMs and hiPSC^W101C^-CMs. Therefore, we measured the XAF-sensitive current in the presence of CCh. The XAF-sensitive current densities in the presence of CCh were not significantly different between both hiPSC-CMs groups (Figure 2D). In addition, the I–V relationships of XAF-sensitive current densities in the presence of CCh in hiPSC^corr^-CMs and hiPSC^W101C^-CMs were virtually overlapping, indicating similar reversal potentials (E_rev_; −77.3 ± 5.0 mV for hiPSC^W101C^-CMs (n = 7) and −74.2 ± 4.1 mV for hiPSC^Corr^-CMs (n = 6)) and amounts of rectification. Thus far, these series of voltage clamp experiments showed that the W101C *KCNJ5* mutation does not affect the total amount of I_K,ACh_ channels or its I–V relationship in hiPSC-CMs but resulted in a more constitutively active I_K,ACh_.

### 2.3. W101C KCNJ5 Results in a Lower Pacemaking Frequency in RA-Treated hiPSC-CMs

**Figure 3:**
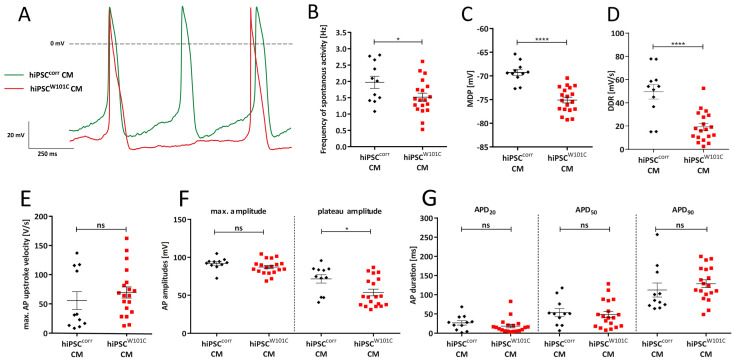
Action potential (AP) parameters under baseline conditions in RA-treated hiPSC^corr^-CMs and hiPSC^W101C^-CMs. (**A**) Typical APs of a hiPSC^corr^-CM and hiPSC^W101C^-CM. (**B**–**F**) Dot plots of spontaneous activity frequency: (**B**) maximal diastolic potential (MDP), (**C**) diastolic depolarization rate (DDR), (**D**) maximal AP upstroke velocity, (**E**) AP amplitudes, (**F**) and AP durations at 20, 50 and 90% of repolarization (APD_20_, APD_50_, and APD_90_; (**G**)). * = *p* < 0.05; **** = *p* < 0.0001; ns = not significant.

Subsequently, the effects of the W101C *KCNJ5* variant on spontaneous activity were evaluated. Figure 3A shows typical action potentials (APs) of a spontaneously beating RA-treated hiPSC^corr^-CM and hiPSC^W101C^-CM under baseline conditions. The average AP parameters are summarized in Figure 3B–F. hiPSC^W101C^-CMs had a slower spontaneous beating activity as indicated by the significant lower frequency of spontaneous APs (Figure 3B). On average, the frequency in hiPSC^corr^-CM and hiPSC^W101C^-CM was 2.0 ± 0.2 Hz and 1.5 ± 0.1 Hz (*p* < 0.05, unpaired *t*-test), respectively. In addition, the maximal diastolic potential (MDP) was about ≈6 mV more negative in hiPSC^W101C^-CMs (−75.1 ± 0.6 mV) compared to the hiPSC^corr^-CMs (−69.3 ± 0.7 mV) (Figure 3C). Furthermore, the diastolic depolarization rate (DDR) was significantly slower in hiPSC^W101C^-CMs (19.3 ± 2.9 mV/s) compared to the hiPSC^corr^-CMs (49.6 ± 6.3 mV/s) (Figure 3D). Other AP parameters were unaffected by the W101C *KCNJ5* variant, except for a lower AP plateau amplitude in hiPSC^W101C^-CMs (Figure 3E–G).

### 2.4. I_K,ACh_ Inhibition Restores Spontaneous Activity in RA-Treated hiPSC^W101C^-CMs

**Figure 4:**
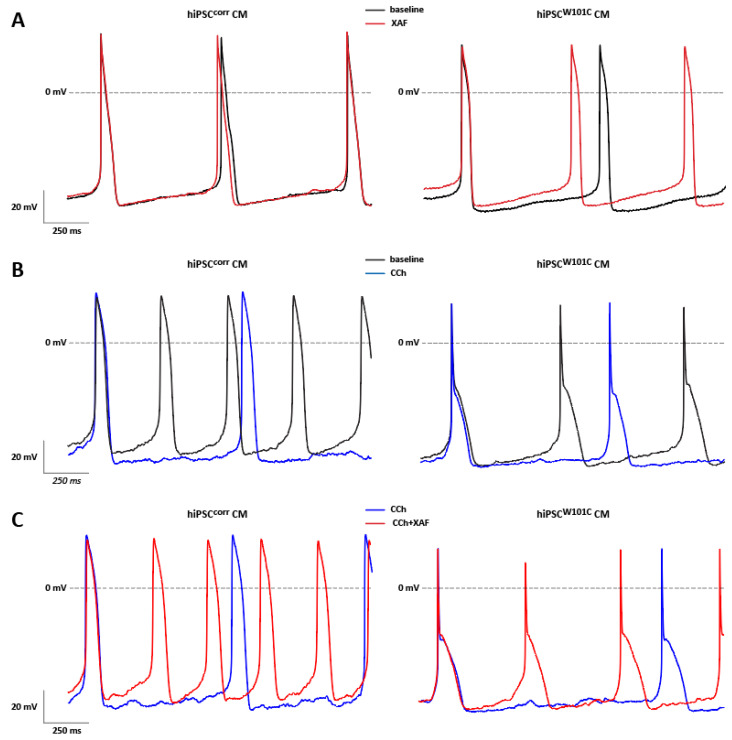
Effects of AP modulating substances in RA-treated hiPSC^corr^-CMs and hiPSC^W101C^-CMs. (**A**) Typical APs of a hiPSC^corr^-CM and hiPSC^W101C^-CM in the absence and presence of XAF. (**B**) Typical APs of a hiPSC^corr^-CM and hiPSC^W101C^-CM in the absence and presence of CCh. (**C**) Typical APs of a hiPSC^corr^-CM and hiPSC^W101C^-CM in the presence of CCh and after additional application of XAF.

Since hiPSC^W101C^-CMs were shown to have a lower spontaneous beat frequency and a more hyperpolarized MDPs (Figure 3), it was tested whether this was due to the constitutively active channel as observed in hiPSC^W101C^-CMs (Figure 2). Therefore, the effects of the I_K,ACh_ blocker XAF-1407 on spontaneous activity in RA-treated hiPSC^corr^-CMs and hiPSC^W101C^-CMs were measured. Figure 4A shows typical APs in the absence and presence of XAF-1407 and the average AP parameters in hiPSC^W101C^-CMs and hiPSC^corr^-CMs are summarized in Supplemental Tables S1 and S2. XAF-1407 resulted in a faster spontaneous or pacemaking activity in hiPSC^W101C^-CMs as indicated by the increased frequency of the spontaneous APs (1.2 ± 0.1 Hz (baseline) vs. 1.7 ± 0.1 Hz (XAF-1407); *p* < 0.001, paired *t*-test; Supplemental Table S1), while it did not affect the pacemaking activity of the isogenic control line hiPSC^corr^-CMs (Figure 4A, Supplemental Table S2). In fact, the frequency of hiPSC^W101C^-CMs in the presence of the channel blocker (1.7 ± 0.1 Hz, n = 9) is now similar to the frequency of the hiPSC^corr^-CMs in the presence of XAF-1407 (1.7 ± 0.3 Hz, n = 3). This indicates that I_K,ACh_ current inhibition in hiPSC^W101C^-CMs restores the frequency of pacemaking. In addition, XAF-1407 also resulted in the depolarization of the MDP in hiPSC^W101C^-CMs (−74.0 ± 0.9 mV (baseline) vs. −72.3 ± 1.0 mV (XAF); *p* < 0.001, paired *t*-test; Supplemental Table S1), without a significant effect on the MDP in hiPSC^corr^-CMs (Figure 4A, Supplemental Table S2). XAF-1407 also significantly decreased the V_max_ in hiPSC^W101C^-CMs (Supplemental Table S1), consistent with the reduced availability of sodium channels in response to MDP depolarization [40]. Other AP parameters were not affected by XAF-1407 in hiPSC^W101C^-CMs and hiPSC^corr^-CMs (Supplemental Tables S1 and S2). Thus, while AP parameters under baseline conditions showed a clear phenotype in hiPSC^W101C^-CMs, this was absent in response to the XAF-1407-induced blockade of the I_K,ACh_ current. Thus, the slow-pacemaking phenotype with hyperpolarized MDPs in RA-treated hiPSC^W101C^-CMs under baseline conditions is important due to the constitutively active GIRK channels.

In voltage clamp measurements (Figure 2), the acetylcholine analog CCh activated a robust I_K,ACh_ current in RA-treated hiPSC^corr^-CMs and hiPSC^W101C^-CMs, but CCh had a tendency to lower I_K,ACh_ density in hiPSC^W101C^-CMs. Next, the effect of 10 µM CCh on spontaneous APs in both hiPSC-CMs groups was tested. In the RA-treated hiPSC^corr^-CMs, CCh caused a significant slowing of spontaneous activity and MDP hyperpolarization (Figure 4B, Supplemental Table S2), consistent with previous findings in RA-treated control lines of hiPSC-CMs findings [32,33]. On average, CCh decreased the frequency of spontaneous activity by approx. 47.6% from 2.1 ±0.2 Hz to 1.1 ± 0.1 Hz (n = 8, *p* < 0.001 (paired *t*-test)), while the MDP increased by about 3.1% from −68.8 ± 0.8 mV to −71.0 ± 0.6 mV (n = 8, *p* < 0.01 (paired *t*-test)) in hiPSC^corr^-CMs (Supplemental Table S2). The AP upstroke velocity (_Vmax_) and AP amplitude (APA) increased significantly due to CCh application, which both well reflect the increased availability of the cardiac sodium channels [40,41] due to the MDP hyperpolarization. CCh did not affect AP durations (APDs) in hiPSC^corr^-CMs (Supplemental Table S2). In the RA-treated hiPSC^W101C^-CMs, CCh induced a small, but significant, slowing of spontaneous activity and MDP hyperpolarization (Figure 4B, Supplemental Table S1). The frequency decreased by approx. 23.5% and the MDP increased by 1.0 ± 0.4%. Other AP parameters were unaffected, likely because the CCh-induced changes in frequency and MDP are rather limited. The application of XAF-1407 in the continued presence of CCh completely abolished the CCh-induced effects in both the hiPSC^corr^-CMs and hiPSC^W101C^-CMs (Figure 4C, Supplemental Tables S1 and S2), indicating that they were mainly due to the activation of I_K,ACh_.

### 2.5. hiPSC^W101C^-Derived Ventricular-like Cardiomyocytes Do Not Show an Electrophysiological Phenotype in Comparison with Controls (hiPSC^corr^-CMs)

**Figure 5:**
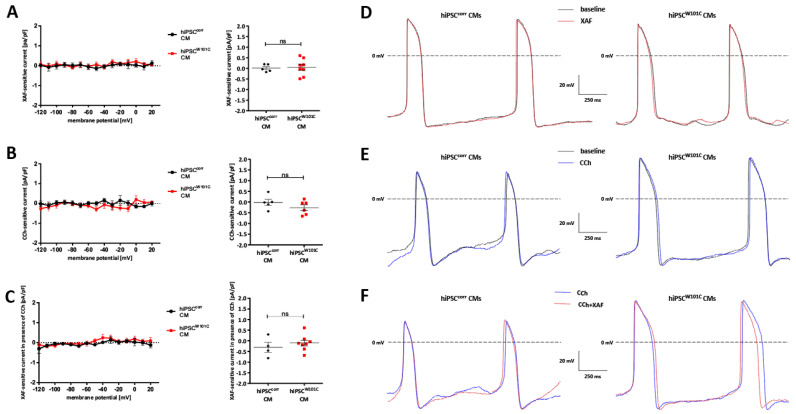
Electrophysiological properties in isogenic untreated, ventricular-like hiPSC^corr^-CMs and hiPSC^W101C^-CMs. (**A**) I–V relationships of XAF-sensitive currents under baseline conditions and dot plots of the XAF-sensitive currents at −120 mV (**right panel**). ns = not significant. (**B**) CCh-sensitive currents and dot plots of CCh-sensitive currents at −120 mV (**right panel**). ns = not significant. (**C**) I–V relationships of XAF-sensitive currents in the presence of CCh and dot plots of XAF-sensitive currents in the presence of CCh at −120 mV (**right panel**). ns = not significant. (**D**) Typical APs of hiPSC^corr^-CM and hiPSC^W101C^-CM in the absence and presence of XAF. (**E**) Typical APs of a hiPSC^corr^-CM and hiPSC^W101C^-CM in the absence and presence of CCh. (**F**) Typical APs of a hiPSC^corr^-CM and hiPSC^W101C^-CM in the presence of CCh and after additional application of XAF-1407.

Although I_K,ACh_ is not functionally present in control ventricular-like hiPSC-CMs [32,42], it could not be excluded that this is also the case in untreated, ventricular-like hiPSC-CMs with the W101C *KCNJ5* variant. Repeating the experiments of Figure 2, but now in ventricular-like hiPSC-CMs, demonstrated that I_K,ACh_ was not present in both ventricular-like hiPSC^W101C^-CMs and hiPSC^corr^-CMs (Figure 5A–C). Consequently, the AP parameters did not differ under baseline conditions, except for a lower V_max_ in hiPSC^W101C^-CMs (Supplemental Tables S3 and S4). Neither XAF, nor CCh and XAF in the presence of CCh altered the AP properties in ventricular-like hiPSC^W101C^-CMs and hiPSC^corr^-CMs (Figure 5D–F, Supplemental Tables S3 and S4). Thus, the W101C *KCNJ5* variant did not induce an altered phenotype in ventricular-like hiPSC-CMs. In addition, because the electrophysiological properties were largely similar in ventricular-like hiPSC^W101C^-CMs and hiPSC^corr^-CMs, additional side effects on other ion channels can be excluded.

## 3. Discussion

As set out in the introduction, the W101C *KCNJ5* variant is associated with bradycardia [9]. To study the exact electrophysiological mechanism underlying the slower pacemaking activity in a human cardiomyocytes model, a patient-derived hiPSC line of a patient carrying the familial pathogenic variant and having corrected the mutation were generated, using CRISPR/Cas9-based gene editing, to create an isogenic control hiPSC line. The hiPSC lines were differentiated into ventricular-like hiPSC-CMs, as well as into atrial-like hiPSC-CMs that robustly expressed GIRK channels using RA-treatment during differentiation (atrial-like CMs). In this study, the focus was on the phenotype of hiPSC-CMs with the W101C *KCNJ5,* and the potential pharmacological rescue by XAF-1407, rather than studying the mechanisms and/or modulation of the constitutively active GIRK due to the W101C *KCNJ5* mutation. However, the studied mutation is located in the first transmembrane domain of GIRK4 [9], which is outside the GIRK4 channels regions such as the N and C terminal known for activation and modulatory binding sites [43,44] or outside the pore region of the GIRK4 channel which is important for selectivity changes [45]. This suggests that the basic modulation of the W101C *KCNJ5* mutant channel will not be affected. Although further studies are required, at least Kir3.1 + Kir3.4W101C complexes in oocytes are still able to respond to M2 receptor stimulation [9] and selectivity is not affected as demonstrated in the present study (Figure 2) and in the work of [9]. Constitutive agonist-independent I_K,ACh_ is also observed in response to atrial fibrillation (AF) [46], and these basal active channels has been extensively studies in the last two decades, as reviewed in detail by Voigt and colleagues [44]. Among others, the novel PKC isoform, PKCε, which is enhanced by AF [47], may cause an increased open channel probability of cardiac GIRK channels by phosphorylation of the channel. The cytosolic C-terminal end of the GIRK4 channel, and especially Ser418, is critical for basal activity and the PKCε-mediated augmentation, as recently demonstrated by Gada et al. [48], thus excluding such a mechanism for the constitutively active GIRK due to the W101C *KCNJ5* mutation. In addition, Logothetis and colleagues created various other mutations in GIRK4 [49,50] and they demonstrated that S143T GIRK4, which is within the pore region of the channel, gives rise to highly active homomeric channels. Although this mutation was used to investigate the individual properties of each subunit of the GIRK4 channel in detail [51,52], the mechanisms of the constitutive active current due to S143T GIRK4 alone were not studied. Kuss et al., [9], however, suggests that the constitutively active GIRK due to the W101C *KCNJ5* have an alteration of the spermidine binding site for W101 *KCNJ5* as cause for the increased I_K,ACh_.

The main findings of the present study are as follows: (1) in RA-treated hiPSC-CMs, the W101C *KCNJ5* variant resulted in a constitutively active I_K,ACh_ current under baseline conditions; (2) CCh application activated I_K,ACh_ in both RA-treated hiPSC^corr^-CMs and hiPSC^W101C^-CMs; (3) the total I_K,ACh_ density was similar in RA-treated hiPSC^corr^-CMs and hiPSC^W101C^-CMs; (4) RA-treated hiPSC^W101C^-CMs had a lower spontaneous (pacemaking) activity due to a more hyperpolarized MDP at baseline conditions; (5) I_K,ACh_ inhibition rescued the spontaneous beating rate in hiPSC^W101C^-CMs; (6) the W101C *KCNJ5* variant did not result in electrical abnormalities in untreated ventricular-like hiPSC^W101C^-CMs. Thus, the present study demonstrates that the familial W101C *KCNJ5* variant results in a slowed pacemaking activity caused by constitutively active GIRK channels. Importantly, the inhibiting of the constitutively active I_K,ACh_ rescues the bradycardic phenotype which is of importance for clinical applications.

Of note, in RA-treated hiPSC-CMs, no significant changes were found in AP repolarization as indicated by the lack of APD changes in response to CCh, although there is a substantial I_K,ACh_ present at positive potentials due to relatively weak inward rectification (Figure 2). The absence of AP repolarization changes is in line with the work of Veerman et al. [22] and Li et al. [23], who found that CCh did not lead to major alterations in APD in RA-treated hiPSC-CMs, except when I_K,ACh_ is largely increased by a *GNB5* variant [32,33]. In contrast, muscarinic receptor activation in human atrial tissue [53,54] and freshly isolated human isolated cardiomyocytes [55] results in an AP shortening. The exact reason for this discrepancy is unknown, but it may be related to the still somewhat immature electrophysiological properties of hiPSC-CMs [56] and potential differences in I_K,ACh_ density between hiPSC-CMs (Figure 2) and human atrial cells [55]. Early- and late-stage development differences in I_K,ACh_ are known due to differences in Kir3.1 and Kir3.4 expression, but our I_K,ACh_ I–V relationship better matches the late-stage than the early-stage development, due to the weaker inward rectification. Further studies using identical experimental protocols are required to solve this issue in detail. Nevertheless, muscarinic receptor activation in freshly isolated rabbit SAN cells does not affect the APD [15], indicating that our used cells’ response is closer to SAN cells than human atrial myocytes. This is supported by the slowing of spontaneously activity in our hiPSC-CMs due to muscarinic receptor activation, which is a hallmark for cardiac pacemaker cells [57].

CCh neither induced an I_K,ACh_ nor affected the pacemaker frequency or MDP in our ventricular-like hiPSC-CMs. These findings are in line with previous studies [32,33,42]. In addition, we found no effects on APD in response to CCh, although it is known that muscarinic cholinergic agonists result in a significant decrease in L-type Ca^2+^ currents I_Ca,L_ in ventricular-like hiPSC-CMs [54]. Consequently, an AP shortening may be assumed; however, it was not present here. The lack of major APD changes is in agreement with other studies [32,33,42,54] and is likely due to the muscarinic-agonists-induced decrease in I_Kr_ [54] and I_Ks_ [58], which limits major changes in the net repolarizing current [54].

In the present study, a limited number of cells was used in some phases of the study, especially when the effects of the I_K,ACh_ blockade and CCh were tested. However, we have performed paired experiments, thus considerably raising the power of statistics at these small numbers of experiments. In addition, the principal findings of the voltage clamp experiments were confirmed by our AP measurements. Furthermore, we found that the E_rev_ of I_K,ACh_ is slightly more positive than the calculated E_K_, in agreement with previous findings in RA-treated hiPSC-CMs [42] and human embryonic stem cell-derived cardiomyocytes [30]. The exact reason is speculative but may be related to a slightly reduced K^+^ selectivity of GRIK channels in hiPSC-CMs. We exclude the immature phenotype of hiPSC-CMs as the reason. Although the ratio between Kir3.1 and 3.4 differs between early- and late-stage development, with consequent differences in rectification, the reversal potential is not different between early- and late-stage development [59]. Nevertheless, because the E_rev_ is similar in RA-treated hiPSC^corr^-CMs and hiPSC^W101C^-CMs (Figure 2D), we exclude mutation-induced loss of ion selectivity, as is shown for some other *KCNJ5* mutations [45]. In addition, RA-treated hiPSC-CMs were used as a surrogate for SAN cells, similar to those employed by Veerman et al. [22]. Very recently, however, differentiation protocols have been developed to create SAN-like hiPSC-CMs [33], but this protocol is not yet commonly used in pacemaker research and was not available at the start of our study. Nevertheless, RA-treated hiPSC-CMs exhibit *HCN4* and spontaneous activity that is sensitive for the pacemaker current (I_f_) blocker, ivabradine [33], and they display I_K,ACh_ suggesting that they are suitable to study pacemaker-related changes [32]. In fact, spontaneous activity changes in response of CCh and I_f_ blockade are largely similar in SAN-like and RA-treated hiPSC-CMs [33], suggesting that our results are not hampered by our used differentiation protocol.

In conclusion, our study shows that the W101C *KCNJ5* mutant—when analyzed in a patient-derived disease model—results in slowed pacemaking by a constitutively active I_K,ACh_ and that I_K,ACh_ inhibition may be useful for clinical applications.

## 4. Materials and Methods

### 4.1. Human iPSC (hiPSC) Generation

The study was approved by the Ethics Committee of the Westfälische Wilhelms University (file number 2010-048-f-s, 2020-302-f-S, 2022-103-f-S). Written informed consent was obtained from the patient.

For the generation of the *KCNJ5*-hiPSC line, PBMCs with the W101C *KCNJ5* mutation were isolated from the patient’s EDTA blood via density gradient centrifugation with Ficoll™ (Sigma-Aldrich, Taufkirchen, Germany). Therefore, the blood plasma was removed by centrifuging the EDTA blood (1000× *g*, 10 min) and the cell pellet was washed with a PBS-buffer mix (DPBS, 2 mM ETDA; 1% BSA) and frozen at −80 °C with Cryo-SFM media until reprogramming.

The Cytotune™-iPS 2.0 Sendai Reprogramming Kit (Thermo Fisher Scientific, Carlsbad, CA, USA) was used for the hiPSC generation process. Cells were seeded for transduction with three Sendai-virus-based vectors (KLF4, SOX2, OCT3/4, c-MYC) as stated in manufacture’s advice. After low-density seeding, clones were picked and cultivated until checking for virus clearance by immunocytochemistry staining (anti-Sendai Rabbit polyclonal antibody; MBL International, Woburn, MA, USA).

### 4.2. Human iPSC Culture

hiPSCs with W101C *KCNJ5* variant were cultured in FTDA medium (DMEM/F12 (Gibco, Life Technologies, Paisley, UK) with 2 mM L-Glutamine (ThermoFisher), 1× ITS (Corning, Bedford, MA, USA), 0.1% HSA (Lucerna-Chem, Lucerne, Switzerland), CD lipid concentrate (Gibco, Life Technologies, Paisley, UK), 50 nM Dorsomorphin (Santa Cruz Biotechnology, Heidelberg, Germany), 2.5 ng/mL Activin A (STEMCELL Technologies, Vancouver, BC, Canada), 0.5 ng/mL TGFß1 (ThermoFisher), 30 ng/mL FGF2 (PeproTech, Cranbury, NJ, USA)) on Matrigel (Corning)- or Geltrex (Thermo Fisher Scientific)-coated plates [60,61]. Cells were examined daily under a light microscope for differentiated sites, density, and morphology.

At 90–100% confluence of the monolayer, hiPSC were passaged with Accutase (Sigma-Aldrich, Taufkirchen, Germany) and 10 µM Y-27632 (Abcam, Amsterdam, Netherlands). For maintenance, 500,000 to 600,000 cells per 6-well plate were laid out in new FTDA medium with ROCK-inhibitor.

### 4.3. hiPSC Editing Using CRISPR/Cas9 (hiPSC^corr^ Generation)

For the in silico design of the crRNA, the Custom Alt-R^®^ CRISP/Cas9 guide RNA design tool was used and the HDR-repair template was designed with the Custom Alt-R^®^ CRISPR-Cas9 guide RNA Design-Tool (https://eu.idtdna.com/site/order/designtool/index/CRISPR_CUSTOM; accessed on 10 August 2019) from Integrated DNA Technologies (IDT, Leuven, Belgium). Additionally, we used CRISPOR (http://crispor.tefor.net/, version 4.95). CRISPOR is a web-based tool which helps to design, evaluate, clone and select guide sequences for the CRISPR/Cas9 system [62,63]. crRNA with a low off-target and high on-target score and specificity towards the mutant allele were used for CRISPR/Cas9-mediated HDR repair (Supplemental Table S5).

First, a duplex with the designed crRNA and tracrRNA was formed and mixed with a ratio of 1:1.2 ALT-R Cas9 enzyme (IDT) for RNP-complex formation according to the manufacturer’s advice. For RNP delivery to the KCNJ5 W101C variant-hiPSC (hiPSC^W101C^) the P4 Primary Cell 4D-Nucleofector™ Kit (Lonza, Cologne, Germany) was used. Therefore, 800,000 hiPSC were harvested with Accutase (Sigma-Aldrich) and 10 µM Y-27632 (Abcam), transferred to a sterile centrifugation tube, centrifuged at 200× *g* (2 min), and the cell pellet resolved in 100 µL of nucleofector solution (Kit P4 Primary Cell 4D-Nucleofector™). Then, 1 µL of ssODN and 1 µL of Alt-R^®^ Cas9 Electroporation Enhancer (IDT) was added to the cells. In total, 4 µL of the previously formed RNP complex was added and the cell suspension was transferred to the nucleofection cuvette. On the 4D nucleofector system (Lonza), the CA-137 program was used. After nucleofection was complete, cells were incubated at 37 °C for 5 min in an incubator, 500 µL of the mTESR plus (STEMCELL Technologies) with Clone R (STEMCELL Technologies) was added, and another 500 µL of medium was laid out on a 12-well plate. After 48 h, cells were detached using Accutase (Sigma-Aldrich) + 10 µM Y-27632 (Abcam) and separated via low-density seeding on a 6-well plate to allow clonal growth. When sufficient cell growth had occurred, the cells were split. Here, one part was laid out on a 24-well plate and the other part was used for DNA isolation and mutation analysis.

### 4.4. Sequencing Analysis

DNA isolation of the young CRISPR/Cas9 clones was carried out based on the protocol of Li et al. [64]. For this purpose, 10 µL of the cell suspension was taken during splitting, centrifuged at 200× *g* for 2 min, and the supernatant was discarded. The cell pellet was resuspended in 5 µL 1× PCR buffer (Thermo Fischer Scientific) containing 1 µL protease K solution [20 µg/µL] (Qiagen, Hilden, Germany) and incubated for 5 h at 56 °C. To inactivate the protease K, the preparation was heated for 15 min at 95 °C in a thermal cycler. The DNA concentration obtained was determined and diluted to approx. 30 ng/µL with AE buffer for subsequent sequencing.

The sequencing reaction was performed using the BigDye^®^ Terminator v3.1. cycle sequencing kit (Applied Biosystems, Life Technologies, Waltham, MA, USA) and specific primer pairs for KCNJ5 (Supplemental Table S5).

### 4.5. Karyotype Analysis

Karyotypes of hiPSC lines were tested with the NanoString nCounter Elements technology with a customized NanoString Probe Set based on nCounter^®^ Human Karyotype Panel following the manufacturer’s advice. In short, 200 ng gDNA was fragmented by AluI digestion. After hybridization with the Capture and Reporter Probe Set at 65 °C, the samples were analyzed on the nCounter^®^ Sprint Profiler (NanoString Technologies, Seattle, WA, USA) and evaluated based on the manufacturer’s protocol for copy number variation (CNV).

### 4.6. Cardiac Differentiation of hiPSC

On the day of differentiation, the confluent monolayer was detached with Accutase + 10 µM Y-27632 and the remaining cell suspension was centrifuged (200× *g*, 2 min). The cell pellet was resuspended in differentiation medium (KO-DMEM (Gibco), 1× Penicillin/Streptomycin/Glutamine (Thermo Fisher Scientific), 5 µg/mL ITS (Corning), 10 µM Y-27632 (Abcam), 200 ng/mL FGF2 (PeproTech), 1 nM CHIR-99021 (STEMCELL Technologies), and 0.75 to 1.0 nM BMP-4 (tested before) (STEMCELL Technologies) [61], and the cell number determined. For KCNJ5-hiPSC lines, 550,000 cells were transferred to a new tube, filled with differentiation medium and mixed with the optimal amount of BMP-4 (tested before) (STEMCELL Technologies), and laid out on matrigel-coated 24-well plates. After 24 h, the differentiation medium was replaced with TS-ASC (KO-DMEM (Gibco), 5.5 mg/L Transferrin (Sigma-Aldrich), 6.75 µg/L Selenium (Sigma-Aldrich), 1× Penicillin/Streptomycin/Glutamine (Thermo Fisher Scientific), and 250 µM ascorbate (Sigma-Aldrich)) [61]. On Day 2 and 3, TS-ASC was supplemented with 0.5 mM C59 (R&D Systems, Minneapolis, MN, USA) for inhibition of the Wnt signaling pathway. Subsequently, the cells were supplied with TS-ASC until the onset of cell contraction.

For atrial cardiac-like cells, 0.5 µM all-trans-retinoic acid (RA; Sigma-Aldrich) was applied during Day 3 and 4 of differentiation to promote the acquisition of atrial-like fate and expression of the GIRK channels [30,31,32]. To exclude the effect of the RA solvent, an equal amount of DMSO was added to the ventricular-like hiPSC-CM during differentiation.

For electrophysiological experiments, hiPSC-CM cultures were enzymatically dissociated into single cells and plated at a low density on glass slides coated with 0.1% gelatine/Matrigel in KO-THAI media (KO-DMEM (Gibco), 1× Penicillin/Streptomycin/Glutamine (Thermo Fisher Scientific), 0.2% human serum albumin (Lucerna-Chem), 250 µM ascorbate (Sigma-Aldrich), 5 µg/mL ITS (Corning), and 0.004% (*v*/*v*) Thioglycerol (Sigma-Aldrich)). All experiments were performed on cells from at least 3 independent differentiation replicates.

### 4.7. Immunofluorescence Staining and Imaging

For the detection of OCT3/4, NANOG, the cells were stained using the Human Pluripotent Stem Cell Marker Antibody Panel Plus from R&D Systems. For the immunofluorescence detection of SOX2, an antibody from Abcam (ab137385) was used.

### 4.8. RT-qPCR

Total RNA was prepared using the Quick-RNA Miniprep- Kit (Zymo Research Corp., Irvine, CA, USA) according to the manufacturer’s instructions. Reverse transcription was performed using the QuantiTect Reverse Transcription Kit from Qiagen according to the manufacturer’s recommendation. The cDNA obtained was stored at −20 °C until use.

For the RT-qPCR, the QuantiTect SYBR Green PCR Kit (Qiagen) or the Luna Universal qPCR Master Mix (NEB, Ipswich, MA, USA) was used according to the manufacturer’s protocol and primers (Supplemental Table S5).

### 4.9. Cellular Electrophysiology in hiPSC-CMs

#### 4.9.1. Data Acquisition

Spontaneously beating hiPSC-CMs showing regular, synchronous contractions were selected for patch-clamp recordings. Membrane currents and APs were recorded with the amphotericin-perforated patch-clamp technique using an Axopatch 200B amplifier (Molecular Devices, Sunnyvale, CA, USA). Signals were low-pass filtered with a cut-off of 5 kHz and digitized at 5 kHz. Voltage control and data acquisition were realized with custom-made software Scope (version 04.04.27; kindly provided by J. Zegers) and analysis was performed with the custom-made software, MacDaq (version 8.0; kindly provided by A. van Ginneken). The experiments were performed at 36 ± 0.2 °C and the potentials were corrected for the calculated liquid junction potential [65]. Cells were superfused with modified Tyrode’s solution containing (in mM): NaCl 140, KCl 5.4, CaCl_2_ 1.8, MgCl_2_ 1.0, glucose 5.5, and HEPES 5.0; pH was set to 7.4 with NaOH. Patch pipettes were pulled from borosilicate glass (Harvard apparatus, UK) and had resistances of 2.0–3.0 MΩ after filling with the pipette solution containing (in mM): K-gluconate 125, KCl 20, NaCl 5, amphotericin-B 0.44, and HEPES 10; pH was set to 7.2 with KOH. Cell membrane capacitance (C_m_) was estimated by dividing the time constant of the decay of the capacitive transient in response to 5 mV hyperpolarizing voltage clamp steps from −40 mV by the series resistance. For membrane current measurements, series resistance was compensated by ≥70%.

#### 4.9.2. Voltage Clamp Experiments

Membrane currents were measured in voltage clamp mode during a descending RAMP protocol from 20 to −120 mV from a holding potential of −40 mV [30,66]. We measured membrane currents sensitive to 100 nM XAF-1407 and 10 µM CCh. Current densities were calculated at a fixed potential during the RAMP, i.e., −120 mV, −110 mV, −100 mV, etc., (Figure 2B) by dividing the current amplitudes by C_m_.

#### 4.9.3. Current Clamp Experiments

APs were recorded in current clamp mode from spontaneously beating single hiPSC-CMs. APs were recorded under baseline conditions and after 4–5 min of application of the various drugs, including 10 µM CCh to activate I_K,Ach_ [30,32] and/or 100 nM XAF-1407 to block I_K,ACH_ [37,38]. APs were characterized by maximal diastolic potential (MDP), maximum upstroke velocity (V_max_), maximum AP amplitude (APA), AP plateau amplitude (APA_plat_ -defined as the potential difference between MDP and potential at 20 ms after the upstroke), and the duration at 20, 50, and 90% repolarization (APD_20_, APD_50_, and APD_90_, respectively). The diastolic depolarization rate (DDR) was measured over the 50 ms time interval starting at MDP + 1 mV. MDP + 1 mV was used rather than MDP because the time at which the MDP + 1 mV was reached could be determined more reliably than the time at which the MDP was reached [67]. The parameter values obtained from 10 consecutive APs were averaged.

### 4.10. Statistics

Data are presented as the mean ± SEM. Statistical analysis was carried out with GraphPad Prism 6 software (GraphPad Software, Inc., Boston, MA, USA). Normality and equal variance assumptions were tested with the Kolmogorov–Smirnov, D’Agostino and Pearson omnibus, or the Shapiro–Wilk test, respectively. For normally distributed data, the (un)paired *t*-test was used and for non-normally distributed data, we used the Mann–Whitney–Rank sum test or the Wilcoxon test. *p* < 0.05 was considered statistically significant.

## Data Availability

Data available on request due to privacy or ethical restrictions.

## Supplementary Materials

The following supporting information can be downloaded at: https://www.mdpi.com/article/10.3390/ijms242015290/s1.
