# Supplementary material for: The W101C KCNJ5 Mutation Induces Slower Pacing by Constitutively Active GIRK Channels in hiPSC-Derived Cardiomyocytes
Source: Int J Mol Sci. 2023 Oct 18;24(20):15290. doi: 10.3390/ijms242015290 (PMC10607318; doi:10.3390/ijms242015290)
Supplement: Supplementary file 1 [file ijms-24-15290-s001.zip › ijms-2645012-supplementary.pdf]

International Journal of
Molecular Sciences

MDPI

Supplement Table S1. Action potential (AP) parameters of atrial (RA-treated) patient-specific hiPSC^{W101C} cardiomyocytes under different experimental conditions.

	Effect of XAF (n=9)		Effect of CCh (n=9)		Effect of XAF in presence of CCh (n=6)	
	**Baseline**	**XAF**	**Baseline**	**CCh**	**CCh**	**CCh+XAF**
**Frequency (Hz)**	1.2±0.1	1.7±0.1 ***	1.7±0.1	1.3±0.2 *	1.5±0.2	2.2±0.2 *
**MDP (mV)**	-74.0±0.8	-72.3±1.0 ***	-76.0±0.9	-76.8±0.9 *	-77.0±1.3	-74.6±0.9 *
**APA (mV)**	92.2±2.7	89.5±3.4	82.2±3.1	78.7±4.4	82.7±3.7	83.4±2.9
**AP Plateau (mV)**	59.9±7.2	60.2±6.8	44.1±3.2	42.4±3.0	39.7±2.7	39.0±2.7
**V_{max} (V/s)**	63.2±12.2	54.7±12.7 *	79.4±15.9	85.6±16.5	108.0±16.4	98.2±13.7 *
**APD_{20} (ms)**	14.6±3.3	16.0±3.5	15.3±4.5	14.1±3.1	10.9±2.1	13.6±7.4
**APD_{50} (ms)**	41.5±9.9	41.5±7.8	48.5±12.6	49.4±15.0	39.5±11.3	47.9±14.1
**APD_{90} (ms)**	118±16.8	117±14.5	138±13.2	136±12.0	124±10.1	146±14.6

Data are mean±SEM. XAF=XAF-1407; CCh=carbachol; MDP=maximal diastolic potential. APA=maximal AP amplitude; AP plateau= AP plateau amplitude measured 20 ms after the AP upstroke; V_{max}= maximal AP upstroke velocity; APD_{20}, APD_{50}, and APD_{90}=AP durations at 20, 50 and 90% of repolarization. * $p<0.05$, *** $p<0.001$.

Supplement Table S2. AP parameters of the RA-treated hiPSC^{corr}-CMs under various conditions.

	Effect of XAF (n=3)		Effect of CCh (n=8)		Effect of XAF in presence of CCh (n=5)	
	**Baseline**	**XAF**	**Baseline**	**CCh**	**CCh**	**CCh+XAF**
**Frequency (Hz)**	1.7±0.2	1.7±0.3	2.1±0.2	1.1±0.1 ***	1.2±0.1	2.4±0.3 *
**MDP (mV)**	-70.5±1.1	-70.5±1.0	-68.8±0.8	-71.0±0.6 **	-70.5±0.9	-68.5±1.2 *
**APA (mV)**	86.6±7.1	86.6±7.2	93.8±1.9	98.7±2.6 *	102.3±2.4	96.3±3.2 *
**AP Plateau (mV)**	76.2±3.4	72.5±2.2	69.8±7.6	75.9±7.0 *	82.2±8.2	78.9±8.5 *
**V_{max} (V/s)**	45.5±35.9	54.7±44.8	59.3±18.0	88.1±21.6 *	87.3±23.5	54.7±17.0 *
**APD_{20} (ms)**	30.7±6.8	23.8±5.8	25.6±7.9	25.9±7.0	25.7±7.3	27.7±6.4
**APD_{50} (ms)**	68.6±18.1	62.7±20.3	48.1±12.6	49.1±11.0	50.5±10.3	51.1±9.6
**APD_{90} (ms)**	104.6±27.3	102.7±29.6	115.4±23.9	88.2±13.6	79.4±7.8	79.0±7.1

Data are mean±SEM. XAF=XAF-1407; CCh=carbachol; MDP=maximal diastolic potential. APA=maximal AP amplitude; AP plateau= AP plateau amplitude measured 20 ms after the AP upstroke; V_{max}= maximal AP upstroke velocity; APD_{20}, APD_{50}, and APD_{90}=AP durations at 20, 50 and 90% of repolarization. * $p<0.05$, ** $p<0.01$, *** $p<0.001$.

Supplement Table S3. AP parameters of ventricular (untreated) isogenic control hiPSC^{corr}-CM under various conditions.

	**Baseline (all cells) (n=10)**	**Effect of XAF (n=4)**		**Effect of CCh (n=6)**		**Effect of XAF in presence of CCh (n=3)**	
		**Baseline**	**XAF**	**Baseline**	**CCh**	**CCh**	**CCh+XAF**
**Frequency (Hz)**	1.2±0.1	1.0±0.1	1.0±0.1	1.3±0.1	1.3±0.1	1.3±0.1	1.4±0.1
**MDP (mV)**	-70.7±0.5	-70.7±0.7	-71.4±0.7	-70.7±0.8	-70.6±0.7	-70.2±0.9	-69.5±1.1
**APA (mV)**	95.1±4.1	102.2±2.9	102.4±2.9	90.4±5.9	92.9±4.6	96.0±5.6	91.1±8.0
**AP Plateau (mV)**	94.6±3.4	103.9±2.5	103.3±2.2	95.9±6.0	95.6±5.3	97.3±7.2	96.4±7.5
**V_{max} (V/s)**	62.5±14.2	99.0±20.3	95.4±16.0	38.1±12.1	37.9±12.0	43.4±22.2	38.3±24.6
**APD_{20} (ms)**	79.1±8.6	87.0±13.7	83.3±14.5	73.7±11.5	74.5±10.4	71.4±12.1	71.7±13.5
**APD_{50} (ms)**	122.4±13.4	141.9±25.3	136.6±26.5	109.5±15.3	111.1±12.8	111.2±16.4	109.6±17.0
**APD_{90} (ms)**	140.0±14.0	162±27.1	156±28.8	125.5±13.8	128.0±12.2	126.6±14.2	124.0±15.6

Data are mean±SEM. XAF=XAF-1407; CCh=carbachol; MDP=maximal diastolic potential. APA=maximal AP amplitude; AP plateau= AP plateau amplitude measured 20 ms after the AP upstroke; V_{max}= maximal AP upstroke velocity; APD_{20}, APD_{50}, and APD_{90}=AP durations at 20, 50 and 90% of repolarization.

Supplement Table S4. Action potential parameters of ventricular (untreated) patient-specific hiPSC^{W101C}-CM.

	Baseline (all cells) (n=23)	Effect of XAF (n=8)		Effect of CCh (n=10)		Effect of XAF in presence of CCh (n=6)	
		**Baseline**	**XAF-1407**	**Baseline**	**CCh**	**CCh**	**CCh+XAF**
**Frequency (Hz)**	1.2±0.1	1.0±0.2	1.0±0.2	1.5±0.2	1.4±0.1	1.5±0.2	1.5±0.2
**MDP (mV)**	-69.7±1.3	-68.7±2.2	-68.8±2.2	-70.2±2.4	-70.1±2.2	-69.6±1.8	-69.5±1.7
**APA (mV)**	98.4±2.2	95.4±2.1	96.8±2.3	102.5±4.3	101.7±4.5	107.1±2.2	106.7±2.1
**AP Plateau (mV)**	93.3±3.2	91.8±2.7	92.6±2.6	95.8±6.9	97.0±6.1	104.9±2.5	104.8±2.5
**V_{max} (V/s)**	23.4±5.4	14.8±2.9	15.5±3.2	35.6±11.1	34.2±10.4	36.7±13.3	34.8±12.4
**APD_{20} (ms)**	91.4±8.0	97.4±17.3	99.3±21.2	84.7±10.8	93.4±10.2	107.6±13.2	106.0±13.0
**APD_{50} (ms)**	129.3±11.2	138.5±23.0	142.8±26.3	118.1±16.0	129.5±14.6	153.8±18.2	150.8±18.2
**APD_{90} (ms)**	152.2±13.2	166.2±29.1	170.2±32.2	135.8±16.8	147.2±15.6	173.1±18.9	169.8±18.9

Data are mean±SEM. XAF=XAF-1407; CCh=carbachol; MDP=maximal diastolic potential. APA=maximal AP amplitude; AP plateau= AP plateau amplitude measured 20 ms after the AP upstroke; V_{max}= maximal AP upstroke velocity; APD_{20}, APD_{50}, and APD_{90}=AP durations at 20, 50 and 90% of repolarization.

Supplement Table S5. Oligonucleotides, crRNA, and HDR-Repair Template

component	Sequence
crRNA [1]	CCTGCCTGTTCTTCGGCTTCATT
HDR-Repair Template [2]	A*A*GTGGCGCTTCAACTT-GCTCGTCTTCACCATGGTTTACACTGTCAC-GTGGCTGTTCTTCGGCTTCATTTGGTGGCT CATTGCTTATATCCGGGGTGAC*C*T
KCNJ5_Ex1-1-up2	CCCTTTCAACACTTAATGTGTAGTTTGC
KCNJ5_Ex1-1-low2	TACCGGTAGGTCTCCTGGACG
KCNJ5_Ex1-2-up2	GGACCCCAAGAAGATTCCAAAACAGG
KCNJ5_Ex1-2-low2	GTCCCGCATGGAGATGACTGC
KCNJ5_Ex1-3-up2	TCCTCTTGGTCCAGGCCATCC
KCNJ5_Ex1-3-low2	CCAGGACTCTCAGGCACAGACC
KCNJ5_Ex02-up2	GATGACTGGATGGATGGATGGATGGATAG
KCNJ5_Ex02-low2	CACACTCAACTATTCCCCTCCCCTG
GAPDH for	ATGACCACAGTCCATGCCATC
GAPDH rev	CGTTCAGCTCAGGGATGACC
HPRT1 for	CCCTTGACTATAATGAATACTTCAGGG
HPRT1 rev	CTGGCGATGTCAATAGGACTCC
CTNT for	AAGGCCAAGGAGCTGTGG
CTNT rev	GGTTATCGTTGATCCTGTTTCGG
KCNJ3 for	TGGGATGACTTGTCAAGCTCG
KCNJ3 rev	GGGAGTAATCAACTTTAAAGAATCCCTC
KCNJ5 for	TGGAAGCCACAGGCATGACC
KCNJ5 rev	GTTGTAGTCCACCTCATAGAAGCCC
RPL37A for	TGGTTCCTGCATGAAGACAGTG
RPL37A rev	TTCTGATGGCGGACTTTACCG
RPS16 for	ATGCTATCCGTCAGTCCATCTCC
RPS16 rev	CCTTCTTGGAAGCCTCATCCAC
NANOG for	GCCTCACACGGAGACTGTC
NANOG rev	GACACTCTTCTCTGCAGAAGTGG
SOX2 for	CTGGCGAACCATCTCTGTGG
SOX2 rev	ACGGTGTCAACCTGCATGG
Oct4 for	CGAGAACCGAGTGAGAGGC
Oct4 rev	ACCACACTCGGACCACATC

1) PAM site (green), nucleotide exchange (red)

2) PAM site (green) with silent mutation

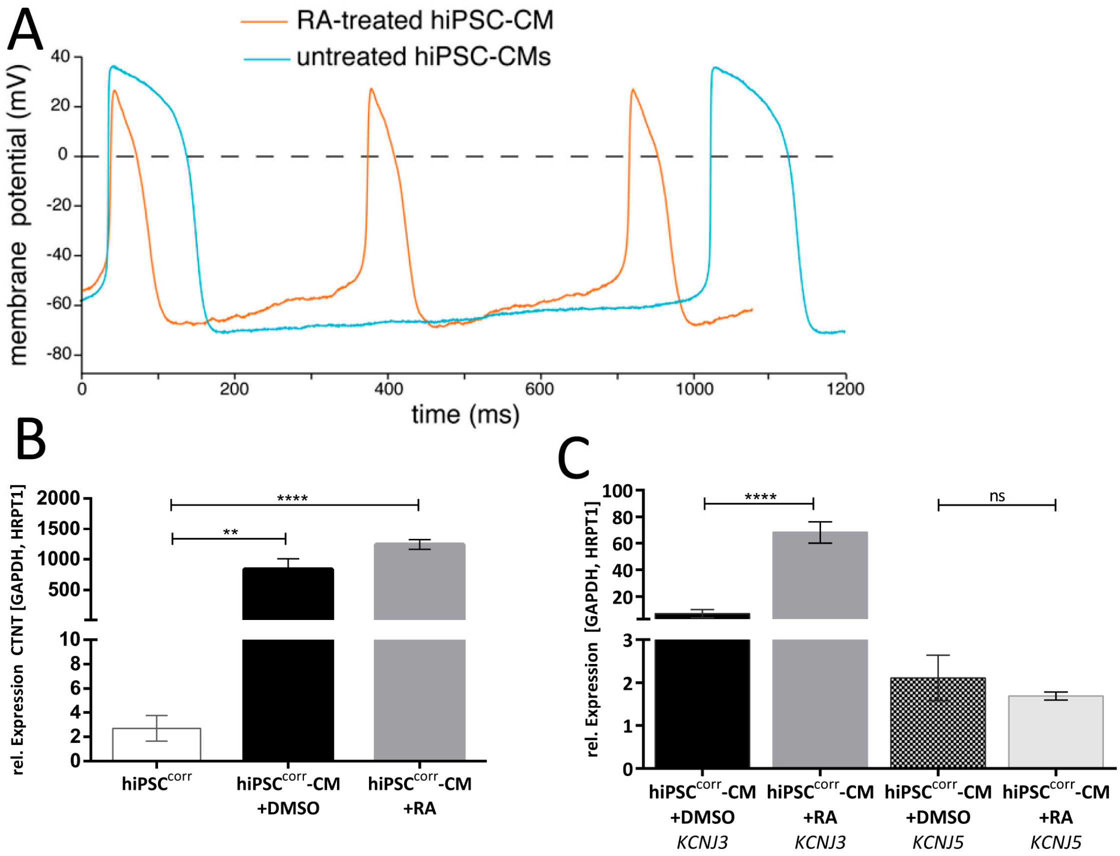

Supplement Figure S1. Application of RA to the differentiation process of hiPSCs results in atrial-like cardiomyocytes (CMs) A, Membrane potential (mV) measurement of hiPSC^{corr}-CM during time, **B**, CTNT expression via RT-qPCR in hiPSC^{corr}, RA-treated hiPSC^{corr}-CMs (atrial) relative to expression in DMSO-treated hiPSC^{corr}-CMs (ventricular), mean±SEM of three biological replicates, normalized to GAPDH and HRPT1, **=p<0.01, ****=p<0.0001, **C**, RNA-transcript expression of genes in RA-treated hiPSC^{corr}-CMs (atrial) relative to expression in DMSO-treated hiPSC^{corr}-CMs (ventricular), mean±SEM of three biological replicates, normalized to GAPDH and HRPT1, ns= not significant, ****=p<0.0001.
